# High Cell Density Cultivation Process for the Expression of Botulinum Neurotoxin a Receptor Binding Domain

**DOI:** 10.3390/toxins14040281

**Published:** 2022-04-14

**Authors:** Alon Ben David, Yoel Papir, Ophir Hazan, Moses Redelman, Eran Diamant, Ada Barnea, Amram Torgeman, Ran Zichel

**Affiliations:** Department of Biotechnology, Israel Institute for Biological Research, Ness-Ziona 7410001, Israel; alonb@iibr.gov.il (A.B.D.); yoelpapier1@walla.com (Y.P.); ophirh@iibr.gov.il (O.H.); mosesred54@gmail.com (M.R.); erand@iibr.gov.il (E.D.); adab@iibr.gov.il (A.B.); amit@iibr.gov.il (A.T.)

**Keywords:** *Clostridium botulinum*, subunit vaccine, recombinant protein expression, fermentation, high cell density cultivation

## Abstract

The receptor-binding domain of botulinum neurotoxin (H_C_ fragment), is a promising botulism vaccine candidate. In the current study, fermentation strategies were evaluated to upscale H_C_ fragment expression. A simple translation of the growth conditions from shake flasks to a batch fermentation process resulted in limited culture growth and protein expression (OD of 11 and volumetric protein yields of 123 mg/L). Conducting fed-batch fermentation with rich media and continuous nutrient supplementation significantly improved culture growth (OD of 40.3) and protein expression (1093 mg/L). A further increase in H_C_ fragment yield was achieved by high cell density cultivation (HCDC). The bacterium was grown in a defined medium and with a combined bolus/continuous feed of nutrients to maintain desired oxygen levels and prevent acetate accumulation. The final OD of the process was 260, and the volumetric yield of the H_C_ fragment was 2065 mg/L, which reflects improvement by an order of magnitude. Purified H_C_ fragments, produced by HCDC, exhibited typical biochemical and protective characteristics in mice. Taken together, the advancements achieved in this study promote large-scale production of the H_C_ fragment in *E. coli* for use in anti-botulism vaccines.

## 1. Introduction

Botulinum neurotoxins (BoNTs) are the most toxic substances known in nature, with an estimated 50% lethal dose (LD_50_) value of 1 ng/kg body weight [1]. BoNTs are mainly produced by the anaerobic bacterium *Clostridium botulinum* and can be classified into at least seven serologically distinct serotypes, of which serotypes A, B, and E are the most abundant in human botulism cases [2]. The toxins are 150-kDa proteins consisting of a 100-kDa heavy chain (H) connected to a 50-kDa light chain (L) by a disulfide bond. All BoNT serotypes share a similar architecture consisting of three structural domains that mediate the intoxication process. The first step of this process includes the attachment of the receptor-binding domain, which is located on the C-terminus of the heavy chain (designated H_C_ fragment), to receptors on neurons and subsequent internalization by endocytosis. The next step involves translocation and release of the light chain into the cytosol, a step considered to be facilitated by the translocation domain found on the N-terminus of the heavy chain. The final step is the cleavage of one of three soluble N-ethylmaleimide-sensitive factor attachment protein receptor (SNARE) proteins by the light chain, which possesses endopeptidase activity, thereby preventing the release of the neurotransmitter acetylcholine from nerve cells into the synapses and thereafter to muscle cell [3,4]. This results in flaccid muscle paralysis and can lead to respiratory failure and eventually death [5].

Currently, the prevention and treatment of botulism are based on the presence of toxin-specific neutralizing antibodies in the circulation. The source of the antibodies can be either self, in the case of prophylactic vaccination, or external, administered as postexposure antitoxin therapy. Vaccines against botulism historically consisted of formalin-inactivated toxins (toxoids) adsorbed to alum hydroxide [3,4,6,7]. Although botulinum toxoid-based vaccines have good safety and efficacy profiles, their manufacturing is complex and costly since it requires large-scale production facilities for this highly hazardous and spore-forming agent. Therefore, efforts to develop botulism vaccines have shifted to recombinant atoxic BoNT-based antigens [8,9,10,11,12]. Among the various antigens examined, the H_C_ fragment is a promising vaccine candidate, as it is rich in neutralizing epitopes. Indeed, a recombinant botulinum vaccine (rBV) composed of the H_C_ fragments of botulinum neurotoxins A and B is under clinical investigation [13,14].

To produce an anti-botulinum vaccine based on H_C_ fragments, an efficient protein expression system is required. The most explored expression systems for recombinant H_C_ fragments are *Escherichia coli* and *Pichia pastoris* [2,7,8,15]. Early attempts to produce recombinant H_C_ fragments were performed using *E. coli* as a host [16,17]. However, as most of the expressed protein was insoluble, subsequent studies have successfully used the methylotrophic yeast [18,19,20,21,22,23]. Nevertheless, since *E. coli* is a more attractive host in terms of genetic manipulation and production processes, more recent efforts have found suitable conditions for H_C_ fragment expression in *E. coli* with yields of up to several dozens of mg per liter of culture [11,15]. We have also previously reported the development of an efficient expression system for the H_C_ fragment of BoNT/A in *E. coli*, which yielded hundreds of milligrams of protein per liter from bacteria grown in shake flasks [24]. For the production of H_C_ fragments as a vaccine candidate, upscaling of the expression process is required. In the current study, various fermentation strategies were evaluated, of which high cell density cultivation (HCDC) resulted in the highest volumetric yield of 2 g of H_C_ fragment per liter of culture.

## 2. Results

### 2.1. Characterization of H_C_ Fragment Expression in Shake Flasks

**Figure 1:**
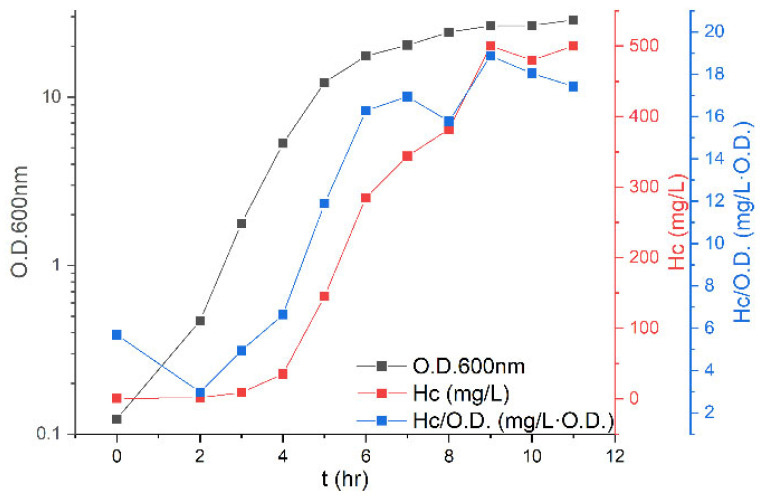
Kinetics of bacterial growth and H_C_ fragment expression in shake flasks. The culture was grown in TB media at 37 °C. Samples were taken during culture growth and used to determine the optical density, the volumetric yield of H_C_ fragment expression, and the specific yield.

The development of the expression system for the receptor-binding domain of botulinum neurotoxin A (the H_C_ fragment) was described previously [24]. Briefly, the expression vector (pET-9a) includes a trxA gene and a codon-optimized gene encoding the H_C_ fragment, with a ribosome binding site dedicated to the H_C_ fragment upstream. The bacterial host is *Escherichia coli* BL21(DE3). The first step toward the development of a fermentation process was the characterization of bacterial growth and H_C_ fragment expression in shake flasks (Figure 1).

The growth of the culture was logarithmic up until 5 h from seeding, with a doubling time of 38 min. Afterward, the growth rate decelerated, and at 9 h after seeding, the culture entered the stationary phase. During the first four hours, the specific yield of the H_C_ fragment was low and in the range of 3–6.6 mg/L·O.D. The specific yield began to increase at the end of the logarithmic phase and reached its highest value as the culture entered the stationary phase (18.9 mg/L·O.D.). At the end of the culture growth, the soluble H_C_ fragment concentration was 500 mg/L.

### 2.2. Upscaling H_C_ Fragment Expression to a 4-L Fermenter

**Figure 2:**
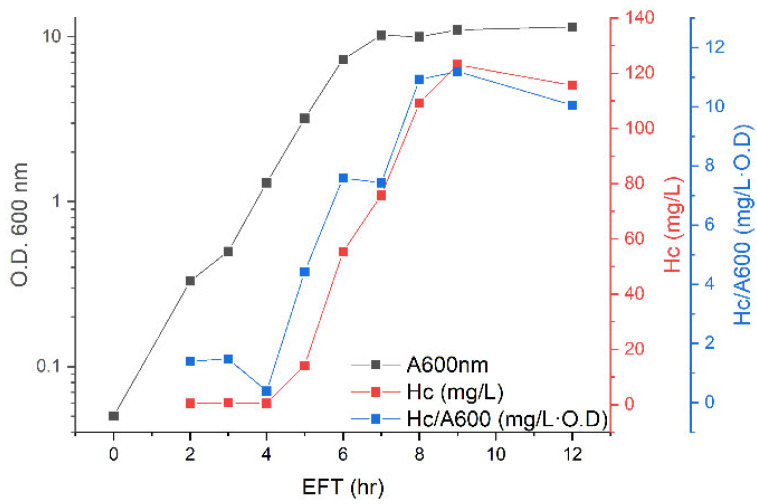
Kinetics of bacterial growth and H_C_ fragment expression using batch fermentation under growth conditions identical to those applied during shake flask experiments. The culture was grown in a 4-L fermenter in TB media at 37 °C.

Next, we upscaled the protein expression process to a 4-L fermenter. Since high expression levels of the H_C_ fragment were achieved using shake flasks, similar growth conditions were first applied. That is, the culture was grown in TB media at 37 °C without the addition of nutrients or adjustment of pH and with the dissolved oxygen level kept above 20% saturation using the fermenter cascade control (Figure 2, Supplementary Table S1). Logarithmic growth was observed during the first 6 h of elapsed fermentation time (EFT), with a doubling time of 50 min, and at 7 h, the culture entered the stationary phase. At the beginning of the logarithmic phase (4 h EFT), the specific yield of H_C_ fragment expression was low (0.4–1.5 mg/L·O.D.) and increased between 4 and 8 h EFT. Despite having similar growth conditions, the final optical density of the culture and volumetric H_C_ fragment expression was considerably lower than those obtained using shake flasks (O.D. of 11.5 and 28.7 and volumetric H_C_ fragment concentrations of 116 and 500 mg/L using the fermenter and shake flasks, respectively). Additionally, the specific yield of H_C_ fragments was lower when using the fermentation process compared to using the shake flasks (11 and 18.9 mg/L·O.D., respectively), implying that the former process did not enable the culture to reach its full potential with regard to growth and protein expression. Therefore, modification of the growth conditions for better suitability to the fermentation process was required.

### 2.3. Fed-Batch Fermentation for H_C_ Fragment Expression

**Figure 3:**
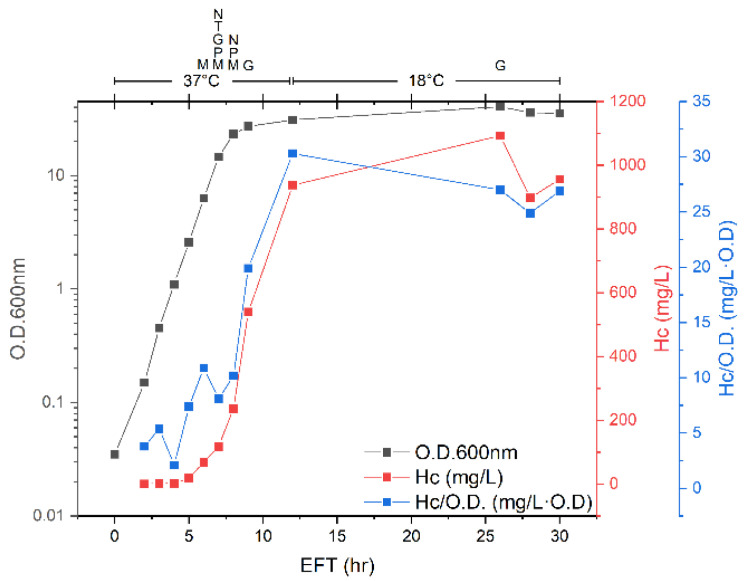
Fed-batch fermentation for H_C_ fragment expression. The growth temperature was set to 37 °C from 0–12 h EFT and 18 °C from 12–30 h EFT. The addition of nutrients is indicated at the top of the graph as follows: M—magnesium sulfate, P—sodium phosphate, G—glycerol, T—trace elements, N—tryptone, and yeast extract concentrate.

To enhance H_C_ fragment expression, the fermentation process was conducted in fed-batch mode, in which nutrients were added to the culture as a function of the accumulated cell mass. The added nutrients were sources of carbon (glycerol), nitrogen and vitamins (tryptone and yeast extract concentrate), magnesium, phosphate, and trace elements. Up until 7 h EFT, the culture growth was logarithmic, with a doubling time of 47 min (Figure 3). At 7 h EFT, the dissolved oxygen decreased to 1.9% saturation at maximal impeller speed and aeration (Supplementary Table S2). Therefore, at this time point, the oxygen uptake rate (OUR) of the bacterium reached the maximal oxygen transfer rate (OTR) of the fermenter, and as a result, the growth rate was reduced. Additionally, the pH of the culture, which showed an upward trend until 7 h EFT, began to decrease, probably due to the accumulation of acetate [25]. To enable further growth without oxygen limitation, we had to lower the OUR of the bacterium. The OUR depends on the cell mass (X), growth rate (µ), and bacterium oxygen yield coefficient (Y_X/O_) (OUR = µ·X/Y_X/O_) [26,27]. To reduce the OUR, the growth temperature of the culture was decreased to 18 °C at 12 h EFT. The temperature decrease nullified the oxygen limitation, as the dissolved oxygen increased and remained above 20%, and at 26 h EFT, the bacterium reached an optical density of 40.5.

The expression of the H_C_ fragment during the fed-batch fermentation process could be divided into four phases. At the beginning of the fermentation (0–4 h EFT), the specific yield of H_C_ fragment was relatively low, in the range of 2.1 and 5.4 mg/L·O.D. Between 5 and 8 h EFT, the specific yield of the H_C_ fragment rose moderately to 7.4–10.9 mg/L·O.D. From 9 to 12 h EFT, the H_C_ fragment concentration increased sharply, and at 12 h EFT, it reached 938 mg/L, with a specific yield of H_C_ fragment of 30.3 mg/L·O.D. This behavior corresponded to the H_C_ fragment expression profile obtained using shake flasks, where the specific yield of the H_C_ fragment increased considerably at the end of the logarithmic growth phase and the beginning of the stationary phase. During the fourth phase of the fed-batch fermentation process, from 12 to 30 h EFT, the H_C_ fragment concentration remained stable.

Upon comparison, the fed-batch fermentation process was superior to the shake flask process in terms of both the specific yield of the H_C_ fragment and the final H_C_ fragment concentration. Additionally, since the change in the H_C_ fragment expression during the fourth phase was negligible, the fermentation process could be accomplished in only 12 h.

### 2.4. High Cell Density Cultivation for H_C_ Fragment Expression

In an attempt to further improve the expression of the H_C_ fragment high cell density cultivation (HCDC) was applied. In HCDC, the culture can reach a very high optical density (>10-fold that of cultivation using shake flasks), which has the potential to result in a high concentration of the desired product. The HCDC enables a decrease in the capital investment required for the construction of production facilities by maximizing the process volumetric yield [25].

An important aspect of growing *E. coli* culture to levels of high cell density is the prevention of acetate production by the bacterium since acetate accumulation can inhibit growth [28,29,30]. Acetate is produced when incomplete oxidation of the substrate occurs. This might occur either under conditions of oxygen limitation or, when sufficient aeration is supplied, under conditions of excess carbon source, especially when the carbon source is glucose [31,32]. Additionally, *E. coli* produces more acetate when grown in a nutrient-rich medium than when grown in a defined medium [30,33].

**Figure 4:**
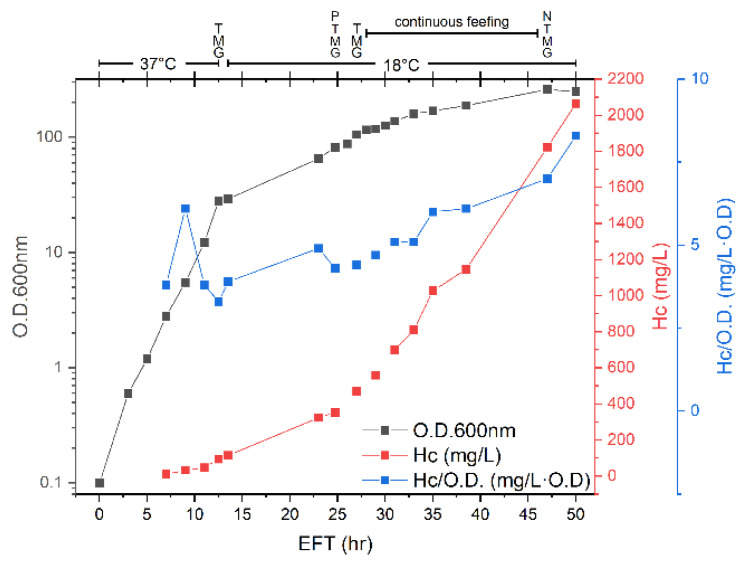
High cell density cultivation for H_C_ fragment expression. The bacterium was grown in a defined medium. At 12.5 h EFT, the temperature was reduced to 28 °C, and at 13.5 h EFT, it was further reduced to 18° C. Nutrient boluses are indicated at the top of the graph as follows: M—magnesium sulfate, P—sodium phosphate, G—glycerol, T—trace elements, N—tryptone and yeast extract concentrate. From 28 h EFT until 47 h EFT, continuous feeding at a constant rate was supplied to limit the oxygen demand of the bacterium and prevent acetate accumulation caused by oxygen limitation.

To prevent acetate accumulation during the fermentation process caused by media composition, a defined medium was used, with glycerol as the carbon source. To prevent acetate accumulation caused by conditions of oxygen limitation, the process was conducted in four phases (Figure 4. Supplementary Table S3). Following inoculation of the fermenter, the temperature was set to 37 °C, and the dissolved oxygen level was kept above 20% saturation using the automatic cascade control. Under these conditions, the growth was logarithmic with a doubling time of 100 min. At 12.5 h EFT, the oxygen demand approached the maximal OTR of the fermenter as the maximal stirring speed was reached. To prevent oxygen limitation, the temperature was then reduced to 28 °C, and at 13.5 h EFT, it was further reduced to 18 °C. The oxygen demand by the bacterium was reduced as a result of the temperature reduction, and the impeller speed decreased accordingly. Between 13.5–28 h EFT, the growth was logarithmic as well, with a much longer doubling time (6.3 h) due to the reduced temperature. During this phase, boluses of nutrients were added to the culture in response to a sudden increase in the dissolved oxygen level due to the complete consumption of a limiting nutrient. The boluses led to an immediate drop in the dissolved oxygen level.

At 28 h EFT, another increase in the dissolved oxygen level was observed. The addition of a nutrient bolus at this point might have resulted in an OUR that was above the maximal OTR of the fermenter since the stirring approached the maximal speed. This could have led to oxygen limitation and thus acetate accumulation and growth inhibition. Therefore, at this point, the process mode was shifted to continuous feeding with a feeding solution that included a carbon source (glycerol), magnesium, and trace elements. The feeding rate was constant and set to limit the growth rate of the bacterium to avoid oxygen limitation (DO > 20%). Under these conditions, the bacterial growth was linear, with an increment of 6.7 O.D. units per hour, which corresponds to the constant flow rate of the substrate into the fermenter. At 47 h EFT, the process was terminated, and an optical density of 260 was observed. Following culture centrifugation, 360 g of wet cells were obtained per liter.

The specific yield of the H_C_ fragment was relatively stable until 13.5 h EFT (approximately 3.8 mg/L·O.D.). From 23 to 47 h EFT, the specific expression yield increased steadily and reached a value of 7 mg/L·O.D. and the volumetric yield reached 1823 mg of H_C_ per liter of culture. Grossmann et al. [34] reported that the expression of recombinant proteins regulated by the T7 promoter is induced spontaneously at the beginning of the stationary phase when the bacterium is grown in rich media. It was also found that higher protein expression levels were obtained in the presence of acetate. Therefore, at the last stage of the fermentation process (47 h EFT), a final bolus of nutrients was supplied, which included glycerol, tryptone, and yeast extract, to simulate growth in rich media and under oxygen limitation. At 50 h EFT, the yield of H_C_ fragment increased to 2065 mg/L, and the specific yield of protein reached 8.3 mg/L·O.D.

### 2.5. Purification and Characterization of H_C_ Fragment Expressed in A HCDC Process

**Figure 5:**
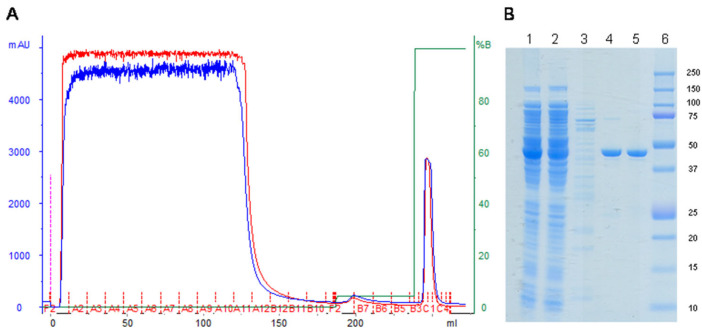
Purification of the H_C_ fragment expressed during the HCDC process. Cells were disrupted by sonication, and the protein was purified from the supernatant by IMAC (**A**) (red and blue lines represent absorbance at 260 and 280 nm, respectively; the green line represents the elution buffer percentage). Samples of the purification process were analyzed by SDS-PAGE (**B**): 1. Disrupted culture supernatant; 2. Flow-through from loading supernatant to IMAC column (unbound proteins); 3. Released impurities at 40 mM imidazole; 4. Purified H_C_ fragment expressed during the HCDC process; 5. Purified H_C_ fragment expressed using shake flasks; 6. Molecular weight marker.

The H_C_ fragment from the HCDC process was purified using IMAC (immobilized metal affinity chromatography) from 20 g of cell paste, which corresponds to 56 mL of culture (Figure 5). A total of 79 mg of purified H_C_ fragment was obtained, which is equivalent to a yield of 1.4 g of purified protein per liter of culture. The H_C_ fragment expressed during the HCDC process migrated in SDS–PAGE with a similar size as the H_C_ fragment expressed using shake flasks (Figure 5B, lanes 4 and 5).

**Figure 6:**
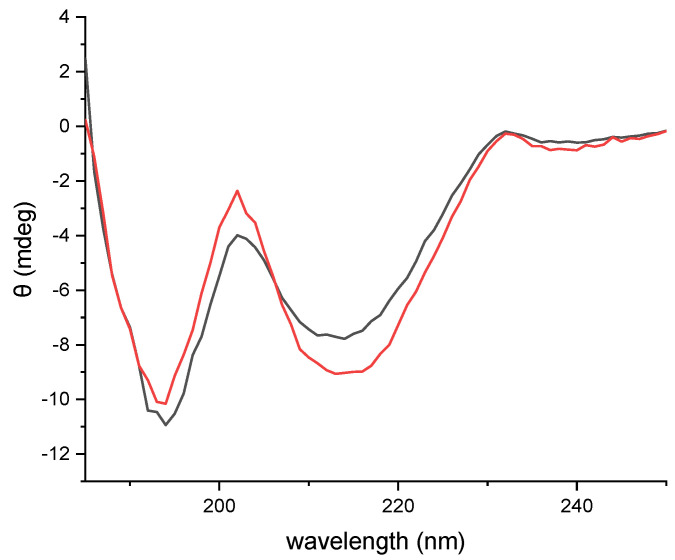
Far-UV CD spectra of H_C_ fragments were expressed using HCDC (black) or shake flasks (red).

We have previously reported the expression and purification of H_C_ fragments expressed in cultures grown in shake flasks and characterized their immunogenicity [24]. The HCDC process differs from the shake flask expression process conditions in terms of media composition, growth temperature, and process duration, as well as the physical running parameters conferred by the fermenter. To test whether these modifications may affect the properties of the expressed H_C_ fragment and its purification process, we compared the structure and immunogenicity of the H_C_ fragment expressed using HCDC to the H_C_ fragment expressed using shake flasks. To evaluate their structural similarity, a circular dichroism (CD) analysis in the far-UV range was conducted (Figure 6). Similar CD spectra were obtained for both proteins, with a negative signal at 194 nm, a local maximum at 203 nm, and a wide negative band between 210 and 220 nm, which is a feature of ß-structures. The spectra of the proteins are also comparable to the spectra of the H_C_ fragment of BoNT/A reported by Tavallaie et al. [35].

The immunogenicity of the H_C_ fragment expressed using HCDC was examined by subcutaneous vaccination of mice (n = 5) with 2 µg of H_C_ fragment adsorbed to alum hydroxide, followed by challenge with 10,000 mice intraperitoneal LD_50_ of BoNT/A after one month. Full protection and no signs of botulism symptoms were obtained in the H_C_ fragment-vaccinated mice, while in a control group of mice injected only with adjuvant, no survival was observed. This result is in accordance with the protective properties reported for the H_C_ fragment in previous studies [15,23,36].

## 3. Discussion

We have previously reported the development of an efficient expression system for the H_C_ fragment of BoNT/A [24]. The system produces the highest H_C_ fragment yields for expression in *E. coli*, and the protein demonstrated good protective immunogenicity, making it an anti-botulism vaccine candidate. In this work, we aimed to upscale the H_C_ fragment expression process from shake flasks to a fermenter. The first stage of our study was to characterize the expression of H_C_ fragments during the shake flask process. The volumetric yield for the H_C_ fragment expressed during this process was 500 mg/L, and the most considerable expression was triggered at the transition of the logarithmic growth phase to the stationary phase, where the specific H_C_ fragment yield reached 18.9 mg/L·O. D (Table 1). This result is in line with the work of Grossman et al. [34], who reported that pET system leaky expression (without the addition of an inducer) is maximal at the beginning of the stationary phase.

Due to the high yield of H_C_ fragments obtained using shake flasks, the goal of our first attempt to upscale the process was to maintain these optimal environmental conditions while using a fermenter. This included a simple translation of the growth in a flask to the fermenter system, i.e., batch growth of the culture in the same media and temperature and without supplements addition. However, the final optical density and H_C_ fragment expression were considerably lower using the batch fermentation process than those obtained using the simple shake flask process (optical densities of 11.5 and 28.7 and H_C_ fragment volumetric yields of 116 and 500 mg/L for the fermenter and shake flask processes, respectively). This behavior of low performance in the fermentation process in comparison to the shake flask process is common, as not all environmental conditions can be maintained upon upscaling, especially physical factors [27]. Therefore, other fermentation strategies were explored. Conducting the fermentation in fed-batch mode resulted in a considerable improvement in all process yield parameters (optical density of 40.5; volumetric H_C_ fragment expression yield of 1093 mg/L; and specific H_C_ fragment expression yield of 30.3 mg/L·O. D) (Table 1). The specific H_C_ fragment yield increased significantly as the culture entered the stationary phase (Figure 3), corresponding to the H_C_ fragment expression profile obtained using shake flasks. To avoid oxygen limitation of the culture, the process temperature was reduced after 12 h of growth to 18 °C. The temperature reduction resulted in an attenuated oxygen uptake rate by the culture and allowed further mass accumulation. However, the additional growth at 18 °C did not contribute significantly to the H_C_ fragment yield, since at 12 h EFT, the specific H_C_ fragment yield reached its maximal value, and the volumetric yield was also close to maximum.

The third fermentation strategy that was explored for H_C_ fragment expression in the current study is HCDC. The advantage of HCDC is the potential for achieving a high volumetric yield through the generation of high cell mass, thereby reducing the fermenter volume required to produce a given amount of recombinant protein. Indeed, the HCDC process yielded the highest volumetric yield of more than 2 g of H_C_ fragment per liter of culture (Table 1). Nevertheless, the specific H_C_ fragment yield of the HCDC process was considerably lower than that of the fed-batch process (maximal specific H_C_ fragment expression yields of 8.3 and 30.3 for the HCDC and the fed-batch processes, respectively, (Table 1)). It should be noted that reduced specific productivity of recombinant protein expression for the HCDC process is precedented [37,38] and can explain why the volumetric yield was only 2-fold higher for the HCDC process than for the fed-batch run, while the optical density was 6.5-fold higher.

The HCDC process reported in this work is unique and simple. In most previous studies reporting the use of HCDC, the prevention of acetate accumulation was accomplished through aeration with pure oxygen and limitation of carbon source concentration using exponential feeding [32,39,40] Our fermentation system setup included standard aeration with compressed air and a simple peristaltic pump for the feeding solution. Thus, the strategy to prevent acetate production due to oxygen limitation was to restrict the oxygen uptake rate of the bacterium rather than increase the oxygen transfer rate of the fermentation system. This was achieved by applying a constant flow rate of nutrients to maintain the dissolved oxygen level above 20% saturation. This strategy to avoid acetate accumulation does not require special adaptations to the fermentation system, making HCDC more attainable.

Since the HCDC process differs significantly from the previously reported shake flask process, it was necessary to test whether the fermenter-produced recombinant protein maintains the same structural and protective properties of the H_C_ fragment expressed during the shake flask process. Therefore, the H_C_ fragment from the HCDC process was purified and characterized. Circular dichroism analysis indicated structural similarity between H_C_ fragments produced by the shake flask and HCDC processes. The CD spectra were also in accordance with CD spectra reported previously for H_C_ fragments [35]. Additionally, full protection was conferred to mice that were vaccinated with H_C_ fragments from the HCDC process and challenged with a lethal dose of botulinum toxin. Therefore, the H_C_ fragment produced by HCDC is properly folded and confers protective immunogenicity.

## 4. Conclusions

The fermentation process developed in this work provided H_C_/A fragment expression yields of >2 g per liter. This value represents an order of magnitude improvement in the expression of this protein in *E. coli* and enables large-scale production of H_C_ fragments as a subunit anti-botulism vaccine. A high yield was achieved by employing a unique HCDC process. The accumulation of acetate in this process was prevented by maintaining the oxygen uptake rate of the bacterium below the maximal oxygen transfer rate of the fermenter, thereby avoiding oxygen limitation. This was accomplished by applying continuous feeding at a constant flow rate that maintained the dissolved oxygen level above 20% saturation. The advantage of this HCDC strategy is that it is less confined by the OTR of the fermenter and it can be carried out with a simple fermentation system without the need for special adaptation, making HCDC more accessible.

## 5. Materials and Methods

### 5.1. Ethics Statement

All animal experiments were performed in accordance with Israeli law and were approved by the Ethics Committee for Animal Experiments at the Israel Institute for Biological Research (protocol No. M-36-2014; approval date 12 June 2014).

### 5.2. Materials

All chemicals were purchased from Merck unless otherwise stated. The yeast extract and tryptone were from Becton, Dickinson, and Company (Franklin Lakes, NJ, USA). Mouse anti-H_C_/A monoclonal antibody and rabbit anti-H_C_/A were described previously [24,41].

### 5.3. Bacteria and Toxin

The H_C_ fragment was expressed in *E. coli* BL21(DE3) harboring the plasmid pET-9a-trx-rbs-H_C_ described previously [24]. *C. botulinum* A was obtained from the Israel Institute for Biological Research collection (strain A198). The sequence of BoNT/A is identical to that of the neurotoxin gene of *C. botulinum* 62A (GenBank accession number M30196). The toxin was prepared from concentrated supernatant of culture grown for 6 days in anaerobic culture tubes.

### 5.4. Expression of the H_C_ Fragment Using Shake Flasks

Starter culture was prepared by inoculating *E. coli* BL21(DE3) carrying the plasmid pET-9a-trx-rbs-H_C_ into a 250 mL Ultra Yield shake flask (Thomson Instrument, Cleveland, CA, USA) containing 40 mL of Luria Bertani media with kanamycin (30 µg/mL). The flask was incubated overnight at 37 °C with agitation (250 rpm). Five milliliters of the starter culture were used to inoculate a 2.5 L Ultra Yield shake flask containing 0.5 L of terrific broth (TB) media with kanamycin (30 µg/mL). The flask was incubated at 37 °C with agitation (250 rpm), and samples were withdrawn during culture growth for optical density measurements and determination of soluble H_C_ fragment concentration in cells using ELISA, as described previously [24].

### 5.5. Fermentation Processes

Fermentation processes were run in a 4-L BioFlo 2000 fermenter (New Brunswick Scientific, Edison, NJ, USA). In all processes, the dissolved oxygen (DO) was set to 20% saturation using the fermenter cascade control, which regulated the impeller speed. Compressed air flow was set manually to 2–10 L/min, depending on culture demand. To achieve a short lag time, the starter cultures were calibrated to be at logarithmic phase (optical density of ~4) upon fermenter inoculation. This was achieved by seeding 10 µL of bacterial glycerol stock into 40 mL of TB media, followed by incubation at 37 °C with agitation (250 rpm) for 16 h. Forty milliliters of starter culture were inoculated into the fermenter. During fermentation processes, samples were withdrawn for optical density measurements using a Pharmacia LKB Ultrospec spectrophotometer and the determination of soluble H_C_ fragment concentration in cells using ELISA, as described previously [24]. For all processes, P-2000 was used as an antifoam agent, and kanamycin was added to a concentration of 30 µg/mL.

#### 5.5.1. Batch-Mode Fermentation

Batch-mode fermentation was run to simulate the H_C_ fragment expression using shake flasks; that is, growth was in TB media at 37 °C, without nutrient supplementation, and without pH adjustments.

#### 5.5.2. Fed-Batch Fermentation

The bacteria were grown in a rich medium containing glycerol (30 g/L), yeast extract (24 g/L), tryptone (12 g/L), NaCl (2 g/L), K_2_HPO_4_ (4.95 g/L), and KH_2_PO_4_ (1.98 g/L). Boluses of nutrients were added as described in the results section. The compositions of the nutrient solution and volumes added (in parenthesis) were as follows: phosphate solution—75 g/L K_2_HPO_4_, 30 g/L KH_2_PO_4_ (100 mL); magnesium solution—50 g/L MgSO_4_ (50 mL); protein concentrate—240 g/L yeast extract, 120 g/L tryptone (100 mL); trace element solution—5 g/L MnSO_4_·H_2_O, 27 g/L FeCl_3_·6H_2_O; 2.6 g/L ZnCl_2_; 1.3 g/L CoSO_4_; 2.2 g/L (NH_4_)_6_Mo_7_O_24_·4H_2_O, 3 g/L CaCl_2_·2H_2_O, 1.85 g/L CuSO_4_·5H_2_O, 2.5 g/L H_3_BO_3_; and 100 mL/l concentrated HCl (2 mL).

#### 5.5.3. High Cell Density Cultivation

The bacterium was grown in a defined medium described by Korz et al. [32]. The fermenter was aerated with compressed air. The pH control was set to 6.8 using an automatic addition of 16% NH_4_OH solution. Feeding solution composition: 730 mL/L glycerol; 1.8 g/L MgSO_4_·7H_2_O; 1.8 mL/L trace element solution. The feeding solution was added using a 101 U/R peristaltic pump (Watson-Marlow, Falmouth, UK) with a MasterFlex 16 tube (MasterFlex, Gelsenkirchen, Germany).

### 5.6. Purification of H_C_ Fragment

Twenty grams of cells from the HCDC process were suspended in a 130 mL binding buffer (20 mM sodium phosphate, 0.5 M NaCl, 20 mM imidazole, pH 7.4) and disrupted by sonication. The cell extract was clarified by centrifugation (14,000× *g*, 30 min) and loaded onto a HisTrap FF 5-mL column (GE Healthcare) mounted on an AKTA Explorer fast protein liquid chromatography system (GE Healthcare). The column was washed with 10 column volumes of binding buffer and 10 column volumes of binding buffer containing 40 mM imidazole. The protein was eluted from the column with elution buffer (20 mM sodium phosphate, 0.5 M NaCl, 500 mM imidazole, pH 7.4). The pure protein was dialyzed against 50 mM sodium phosphate and 50 mM NaCl (pH 6.5) and stored at −70 °C. The concentration of the pure H_C_ fragment was determined at 280 nm using a NanoDrop spectrophotometer (Thermo Scientific, Waltham, MA, USA) with the following parameters: molecular weight, 50,519 g/mol; extinction coefficient, 86,250 cm^−1^ M^−1^ (calculated using the Peptide Properties Calculator [www.basic.northwestern.edu/biotools/proteincalc.html accessed on 12 April 2011]).

### 5.7. Circular Dichroism Analysis

Circular dichroism (CD) analysis was conducted using a Chirascan spectrometer (Applied Photophysics, Leatherhead, UK) at Bar-Ilan University. Spectra were measured from 185 to 400 nm in 1 nm steps and a bandwidth of 3.7 nm. Samples were diluted to 2–4 µM and analyzed in a 1 mm quartz cuvette. Sodium phosphate (50 mM) and NaCl buffer (50 mM) were used as blanks.

### 5.8. Mouse Vaccination and Toxin Challenge

The pure H_C_ fragment from the HCDC process was diluted with phosphate-buffered saline and adsorbed to aluminum hydroxide [final concentration of Al(OH)_3_, 0.5% (wt/vol)] to obtain 2 µg protein per injection dose (0.5 mL). The vaccine preparation was injected once subcutaneously into 5 mice (CD-1; Charles River, UK) per group. The mice were challenged with 10^4^ MsLD_50_ after 21 days. Survival was monitored for 7 days.

## Figures and Tables

**Table 1:** Comparison of H_C_ fragment expression processes.

	Shake Flask	Batch Fermentation	Fed-Batch Fermentation	HCDC
OD *	28.7	11	40.5	250
the volumetric yield of H_C_ fragment (mg/L) *	500	123.1	1093	2065
specific yield of H_C_ fragment (mg/L·OD) *	17.4	11.2	27	8.3
process duration (h) *	11	11	26	50

* Values for EFTs with the maximal volumetric yield of H_C_ fragment expression.

## Data Availability

The datasets generated for this study are available on request to the corresponding author.

## Supplementary Materials

The following supporting information can be downloaded at: https://www.mdpi.com/article/10.3390/toxins14040281/s1, Table S1: Process data of batch fermentation for H_C_ fragment expression in 4-L fermenter applying shake flasks growth conditions. The culture was grown in TB media at 37 °C. Table S2: Process data for fed-batch fermentation for H_C_ fragment expression. Table S3: Process data for high cell density cultivation for H_C_ fragment expression.
