# Supplementary material for: High Cell Density Cultivation Process for the Expression of Botulinum Neurotoxin a Receptor Binding Domain
Source: Toxins (Basel). 2022 Apr 14;14(4):281. doi: 10.3390/toxins14040281 (PMC9031141; doi:10.3390/toxins14040281)
Supplement: Supplementary file 1 [file toxins-14-00281-s001.zip › toxins-1669775-supplementary.pdf]

Supplementary Materials: High Cell Density Cultivation Process for the Expression of Botulinum Neurotoxin A Receptor Binding Domain

Alon Ben David, Yoel Papir, Ophir Hazan, Moses Redelman, Eran Diamant, Ada Barnea, Amram Torgeman and Ran Zichel

Table S1. Process data of batch fermentation for H_C fragment expression in 4-L fermentor applying shake flasks growth conditions. Culture was grown in TB media at 37 °C.

EFT (hr)	O.D.600nm	pH	DO (%)	Impeller speed (rpm)	Air flow (L/min)	Volumetric H_C fragment expression yield (mg/L)	Specific H_C fragment expression yield (mg/L·O.D)
0	0.05	7.25	88	150	2		
2	0.33	7.21	31	150	2	0.5	1.40
3	0.5	7.18	25	227	2	0.7	1.47
4	1.3	7.14	24	345	4	0.5	0.40
5	3.2	7.12	25	525	5	14.2	4.43
6	7.3	7.3	24	847	6	55.4	7.59
7	10.2	7.68	26	812	7	75.8	7.43
8	10	7.78	26	721	7	109.3	10.93
9	11	7.91	25.3	710	7	123.1	11.19
12	11.5	8.29	25.3	197	7	115.6	10.05

Toxins **2022**, *14*, 281. https://doi.org/10.3390/toxins14040281 www.mdpi.com/journal/toxins

Table S2. Process data for fed-batch fermentation for H_C fragment expression.

EFT (hr)	O.D.600nm	pH	DO (%)	Impeller speed (rpm)	Air flow (L/min)	Volumetric H_C fragment expression yield (mg/L)	Specific H_C fragment expression yield (mg/L·O.D)	Supplements*
0	0.035	6.96	95	150	2			
2	0.15	7	43	150	2	0.6	3.8	
3	0.455	6.96	23.1	206	2	2.4	5.4	
4	1.1	6.93	23	330	3	2.3	2.1	
5	2.6	7	22	490	3	19	7.4	
6	6.3	7.23	22	810	4	69	10.9	M
7	14.6	7.65	1.9	1000	10	118	8.1	M,P,G,T,N
8	23.2	7	0	1000	10	236	10.2	M,P,N
9	27.2	7	0.3	1000	10	540	19.9	G
**12	31	7	25	990	10	938	30.3	
26	40.5	8.25	20	340	5	1093	27.0	G
28	36	7.6	20	494	5	898	24.9	
30	35.5	7.45	20.2	470	5	956	26.9	

* Supplements: M - $MgSO_4$, P - sodium phosphate, G - glycerol, T - trace elements, N - tryptone and yeast extract concentrate. ** temperature was set to 18°C.

Table S3. Process data for high cell density cultivation for Hc fragment expression.

EFT (hr)	O.D.600nm	pH	DO (%)	Impeller speed (rpm)	Air flow (L/min)	Volumetric Hc fragment expression yield (mg/L)	Specific Hc fragment expression yield (mg/L·O.D)	Supplements*
0	0.1	6.83	89	200	2			
3	0.6	6.84	20	229	2			
5	1.2	6.82	20	284	2			
7	2.8	6.8	19	377	2	11	3.8	
9	5.5	6.8	17.5	522	2	33	6.1	
11	12.2	6.8	18.2	751	5	47	3.8	
**12.5	28	6.8	20	1000	10	94	3.3	G,M,T
***13.5	29.2	6.8	20	420	10	115	3.9	
23	65.5	6.8	19.9	660	10	324	4.9	
24.75	82	6.8	18.7	768	7	354	4.3	G,M,T,P
26	88	6.8	20	876	8			
27	106	6.8	19.7	922	10	470	4.4	G,M,T
28	116	6.8	18.3	927	10			Continuous feeding of G, M,T
29	118	6.8	18	1000	10	559	4.7	
30	126	6.8	23.2	909	10			
31	138	6.8	20.6	861	10	698	5.1	
33	160	6.8	20.5	894	10	811	5.1	
35	170	6.8	20	926	10	1028	6.0	
38.5	189	6.8	18.7	1000	10	1147	6.1	
47	260	6.8	12	1000	10	1823	7.0	G,M,T,N
50	250	8	20	1000	10	2065	8.3	

*Supplements: M - $MgSO_4$, P - sodium phosphate, G - glycerol, T - trace elements, N - tryptone and yeast extract concentrate. **temperature was set to 28°C. ***temperature was set to 18°C.
